# A Systematic Review of Visual Impairment and Cognitive Decline Among Older Adults

**DOI:** 10.1093/geroni/igaa057.1707

**Published:** 2020-12-16

**Authors:** Niranjani Nagarajan, Bonnielin Swenor, Lama Assi, Joshua Ehrlich, Heather Whitson

**Affiliations:** 1 Johns Hopkins University School of Medicine, Baltimore, Maryland, United States; 2 Johns Hopkins University, Baltimore, Maryland, United States; 3 Johns Hopkins Wilmer Eye Institute, Baltimore, Maryland, United States; 4 University of Michigan, Ann Arbor, Michigan, United States; 5 Duke University, Durham, North Carolina, United States

## Abstract

Cognitive and visual impairments frequently coexist. With the aging of populations worldwide, the prevalence of these conditions are projected to increase substantially over time. A number of studies suggest that cognitive function and vision impairment are associated, and it is hypothesized to be due to a (1) common cause etiology, where both share common risk factors, and/or (2) causal association, where visual impairment causes cognitive decline. Sensory loss can lead to increased cognitive load, structural and functional changes in the brain, and/or decreased emotional, social, and physical well-being, all of which could potentially increase the risk of cognitive impairment. We conducted a systematic review of the existing literature, examining the association between cognitive and visual impairment among older adults. A total of 80 observational studies that reported a measure of association between visual and cognitive function and met the following criteria were included: (1) cross-sectional or longitudinal study design, (2) baseline mean age of participants ≥50 years, and (3) sample size of ≥100 participants. Of these 80 studies, 56 found a positive, significant association between visual function and cognitive decline. Forty-nine of the 56 studies used objective measures to test for visual acuity, contrast sensitivity, or visual fields. The sample included participants from 14 countries, including the US, UK, China, and Australia among others. Converging evidence of an association between vision impairment and subsequent cognitive decline suggests that visual impairment is a possible modifiable risk factor for cognitive decline and dementia. This hypothesis should be tested in prospective, controlled studies.

